# Supplementary material for: Activation of c-Jun by human cytomegalovirus UL42 through JNK activation
Source: PLoS One. 2020 May 5;15(5):e0232635. doi: 10.1371/journal.pone.0232635 (PMC7199950; doi:10.1371/journal.pone.0232635)

Original images for immunoblots presented in Figure 4A

c-Jun

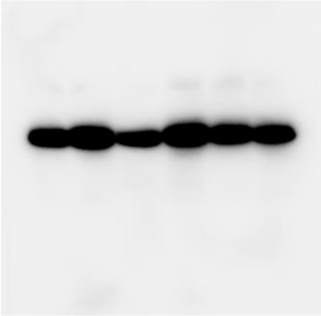

JNK

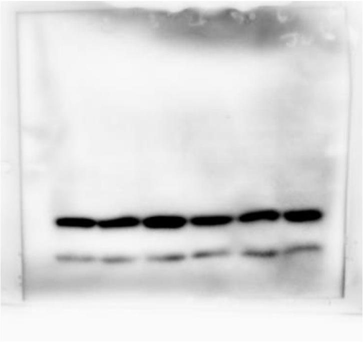

HA

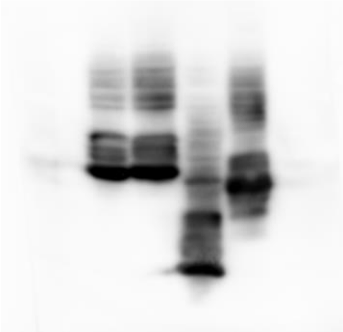

p-c-Jun

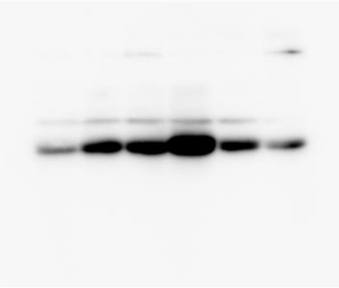

p-JNK

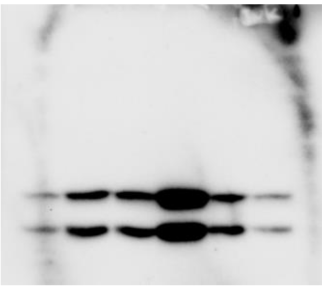

actin

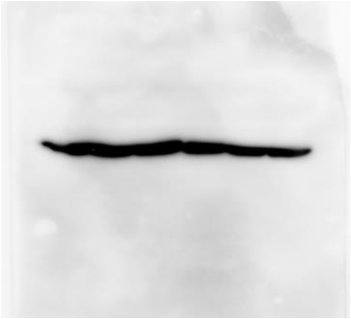

Itch

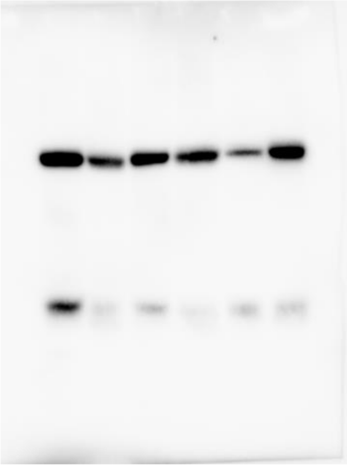

Original images for immunoblots presented in Figure 4B

c-Jun

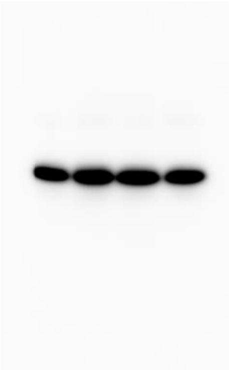

JNK

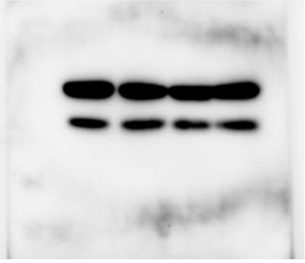

EGFP

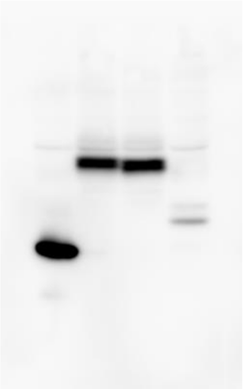

p-c-Jun

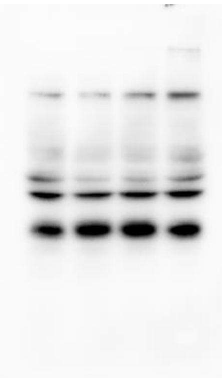

p-JNK

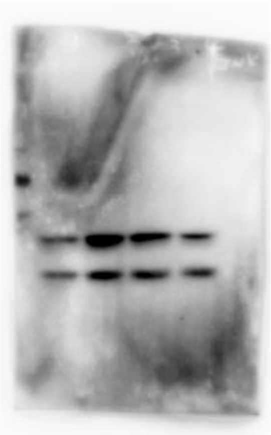

actin

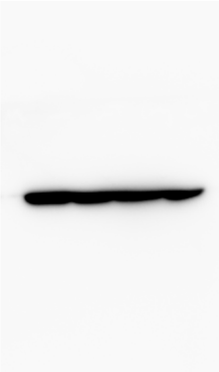

Itch

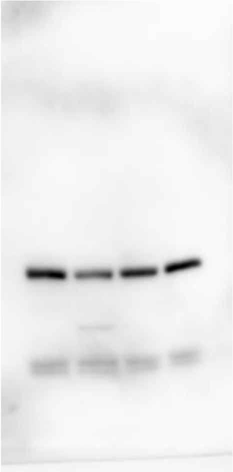

Original images for immunoblots presented in Figure S2

UL42

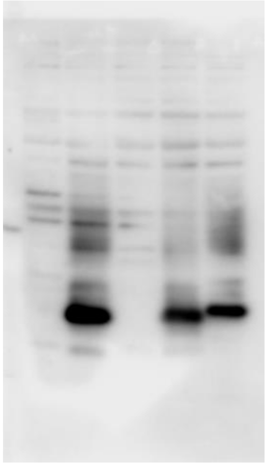

c-Jun

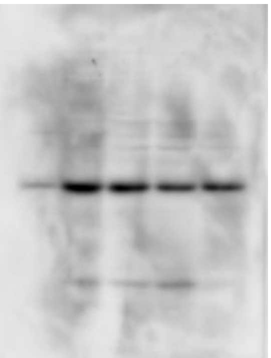

p-c-Jun

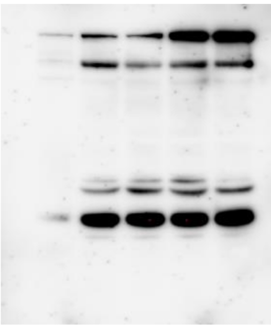

actin

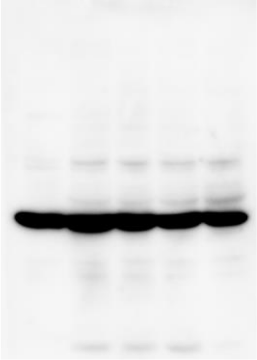

Supplement: S1 Data — (PDF) [file pone.0232635.s001.pdf]
