# Supplementary material for: Activation of c-Jun by human cytomegalovirus UL42 through JNK activation
Source: PLoS One. 2020 May 5;15(5):e0232635. doi: 10.1371/journal.pone.0232635 (PMC7199950; doi:10.1371/journal.pone.0232635)
Supplement: S1 Table — (DOCX) [file pone.0232635.s002.docx]

S1 Table. Primer list

| Primer # | Primer name | Sequence (5'->3') | Purpose |
| --- | --- | --- | --- |
| P1 | HArev | agcgtaatctggaacatcgtatggg | Inverse PCR |
| P2 | UL42(fw151-165)-inverse | ctggttagttcgccgtcgccgcgac |  |
| P3 | UL42(rev150-133)-BglII | gggagatctgcagtacgggggtcgata |  |
| P4 | UL42dTMDrvXhoI(+) | ggggctcgagttaccatttccagccgttg | Inverse PCR and the pEGFP-UL42Ct construction |
| P5 | HCMVUL42fwBam | ggggatccatggagcccacgccgatgc | The construction for pEGFP plasmids. |
| P6 | UL42rv(+)XhoI | ggggctcgagttatcccgatgttgacaccgtcccc |  |
| P7 | UL42TMDfw-BglII | ggggagatctacttttgctctcttag |  |
| P8 | HCMVUL42AY1fw | gacgcggccgccacctacgagcaggcca | To construct pEGFP-UL42ay |
| P9 | HCMVUL42AY1rv | ggtggcggccgcgtcgtcgtgatcccg |  |
| P10 | HCMVUL42AY2fw | cagcgcagcggcctatcgacccccgtactg |  |
| P11 | HCMVUL42AY2rv | taggccgctgcgctgcagtcaggtggtgg |  |
| P12 | aphAI rv MluI | ggacgcgtgccagtgttacaaccaattaacc | To amplify Km^r^ cassette and construct I-SceI-Kmr-UL42 fragment |
| P13 | HCMVUL42up50+ins50+K fw MluI | ggacgcgttcggtaccgtctccaccacagttaccaccgtcgccgtcactgccaccgacatggagcccacgccgatgctccgcgaccgggatcacgacgacgcgccccctagggataacagggtaatcgattt |  |
| P14 | HCMV UL42dn50+25mer rv | tattccgtagcagcaatgatggtacagtcaagcacatgatctatttcccgttatcccgatgttgacaccgtcccc | To amplify I-SceI-Km^r^-UL42 fragment |
