# Supplementary material for: Activation of c-Jun by human cytomegalovirus UL42 through JNK activation
Source: PLoS One. 2020 May 5;15(5):e0232635. doi: 10.1371/journal.pone.0232635 (PMC7199950; doi:10.1371/journal.pone.0232635)
Supplement: S1 Fig — The luciferase assay results of three independent experiments are shown. A plasmid expressing the indicated UL42 mutant tagged with HA (A) or with EGFP (B), the luciferase reporter plasmid pAP1(PMA)-TA-Luc, and the control plasmid pRL-TK were transfected into HEK293T cells. Ratios of firefly luciferase activities to Renilla luciferase activities obtained in triplicated wells are shown as the means ± SEMs. (A) pCAGGS (Vec), pCAGGS-HAUL42WT (WT), -HAUL42PA (PA), -HAUL42ΔN (ΔN), and -HAUL42ΔI (ΔI). (B) pEGFP-C1 (Vec), -UL42WT (WT), -UL42AY (AY), and -UL42Ct (Ct). (PPTX) [file pone.0232635.s003.pptx]

## Slide 1
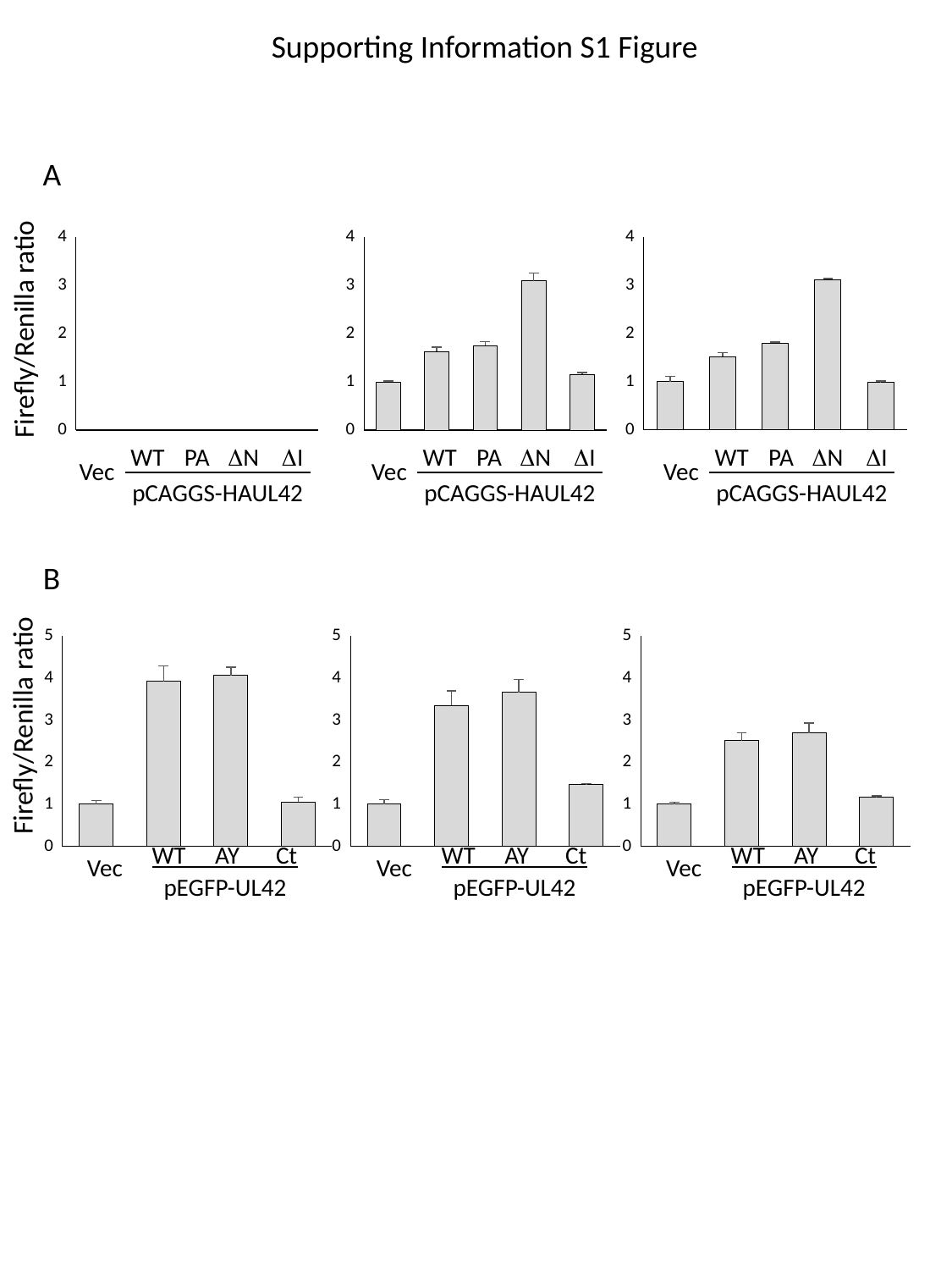

Supporting Information S1 Figure
A
### Chart
| Category | |
|---|---|
### Chart
| Category | |
|---|---|
### Chart
| Category | |
|---|---|Firefly/Renilla ratio
WT
PA
DN
DI
Vec
pCAGGS-HAUL42
WT
PA
DN
DI
Vec
pCAGGS-HAUL42
WT
PA
DN
DI
Vec
pCAGGS-HAUL42
B
### Chart
| Category | |
|---|---|
### Chart
| Category | |
|---|---|
### Chart
| Category | |
|---|---|Firefly/Renilla ratio
WT
AY
Ct
Vec
pEGFP-UL42
WT
AY
Ct
Vec
pEGFP-UL42
WT
AY
Ct
Vec
pEGFP-UL42
