# Supplementary material for: Activation of c-Jun by human cytomegalovirus UL42 through JNK activation
Source: PLoS One. 2020 May 5;15(5):e0232635. doi: 10.1371/journal.pone.0232635 (PMC7199950; doi:10.1371/journal.pone.0232635)
Supplement: S2 Fig — HA-tagged UL42 proteins (A) and EGFP-tagged UL42 proteins (B) were expressed in HEK293T cells and analyzed with immunofluorescence assay. A. The cells expressing HA-tagged UL42 derivatives were stained with anti-HA (red) and anti-c-Jun (green) antibodies. The nuclei were stained with DAPI (blue). B. The cells expressing EGFP-tagged UL42 derivatives were reacted with anti c-Jun antibody and then with Alexa Flora 647-conjugated secondary antibody (red). EGFP fluorescence and nuclei staining with DAPI are shown in green and blue, respectively. Bar = 10μm. (PPTX) [file pone.0232635.s004.pptx]

## Slide 1
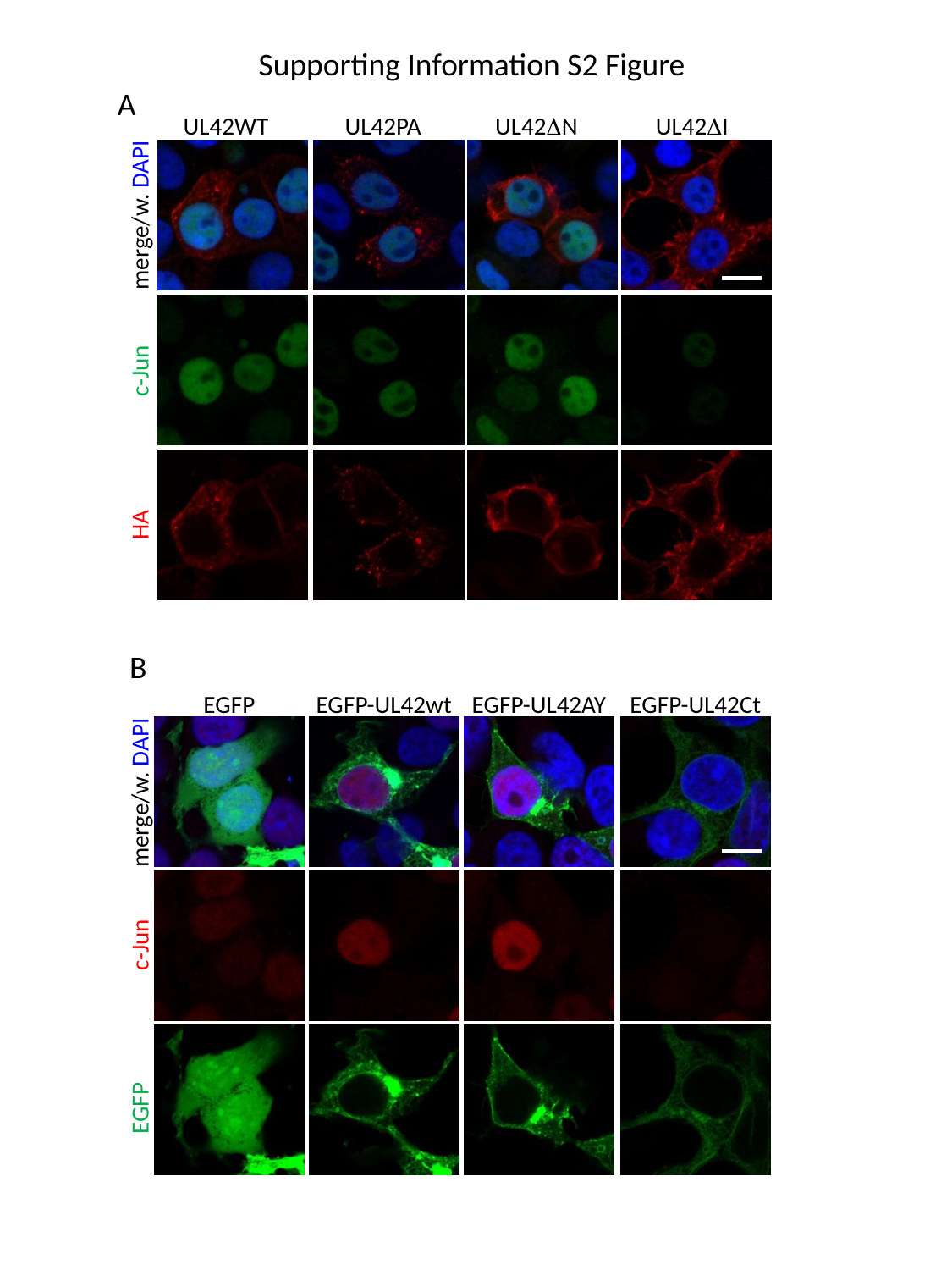

Supporting Information S2 Figure
A
UL42WT
UL42PA
UL42DN
UL42DI
merge/w. DAPI
c-Jun
HA
B
EGFP
EGFP-UL42wt
EGFP-UL42AY
EGFP-UL42Ct
merge/w. DAPI
c-Jun
EGFP
