# Supplementary figures and images for: Activation of c-Jun by human cytomegalovirus UL42 through JNK activation
Source: PLoS One. 2020 May 5;15(5):e0232635. doi: 10.1371/journal.pone.0232635 (PMC7199950; doi:10.1371/journal.pone.0232635)

## Slide 1
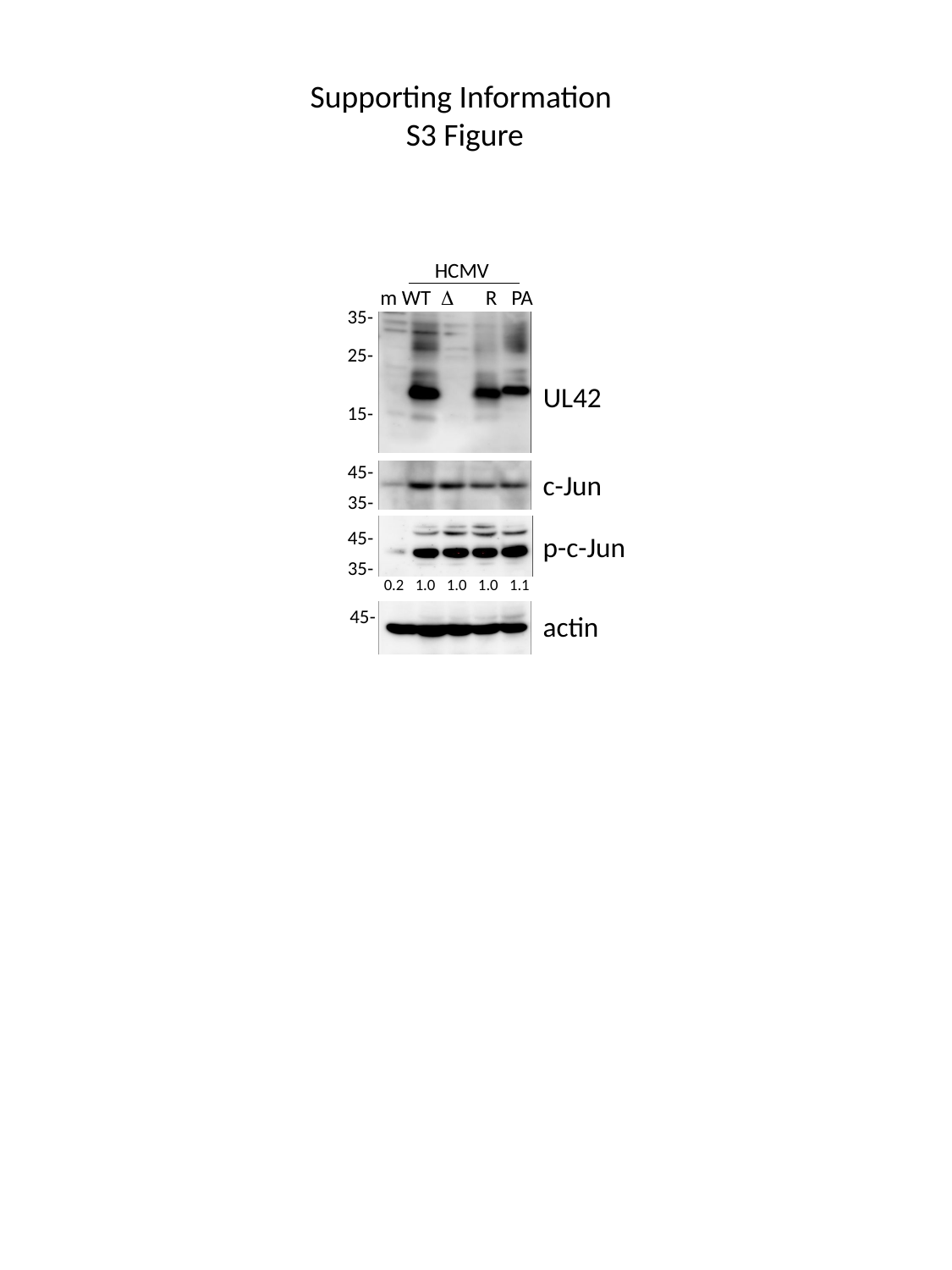

Supporting Information
S3 Figure
HCMV
m WT D R PA
35-
25-
UL42
15-
45-
c-Jun
35-
45-
p-c-Jun
35-
| 0.2 | 1.0 | 1.0 | 1.0 | 1.1 |
| --- | --- | --- | --- | --- |
45-
actin

Supplement: S3 Fig — Fibroblasts, hTERT-BJ1 cell- were mock infected (m) or infected with the following HCMV strains at a multiplicity of infection (MOI) of 5, harvested at 3 day post-infection, and their lysates were analyzed by immunoblotting with the indicated antibodies. WT: HCMV encoding wild-type UL42, R: HCMV encoding rescued UL42, PA: HCMV encoding UL42PA, Δ: HCMV lacking UL42. (PPTX) [file pone.0232635.s005.pptx]
