# Supplementary material for: Activation of c-Jun by human cytomegalovirus UL42 through JNK activation
Source: PLoS One. 2020 May 5;15(5):e0232635. doi: 10.1371/journal.pone.0232635 (PMC7199950; doi:10.1371/journal.pone.0232635)
Supplement: Attachment — Submitted filename: Review Report Koshizuka.pdf [file pone.0232635.s006.pdf]

The manuscript by Koshizuka and Inoue shows a novel role for the human cytomegalovirus (HCMV) protein UL42, activating AP-1 via c-Jun and JNK phosphorylation. The authors have previously reported that this viral protein interacts with the ubiquitin E3 ligase Itch, degrading it. They now show, using UL42 mutants, that the functional domain employed by this viral protein to regulate c-Jun activation does not map to the two PPXY motifs responsible of the interaction with Itch, but instead it involves a region containing the C-terminal TMD. Overall, the study reports an interesting observation and suggests a potential contribution of UL42 to viral replication via activation c-Jun/JNK.

However, a mayor problem of the study is the minimal amount of data presented, which in addition do not provide information on the mechanism by which UL42 activates c-Jun and JNK. Another mayor issue lies on the fact that all the observations described in the study are based on the UL42 protein ectopically expressed, and there are not indications that these UL42-mediated processes occur in the context of the HCMV infection. Although HCMV encodes additional proteins that activate the JNK pathway, it would seem important to determine the effect of a HCMV defective in UL42, or more specifically of HCMVs with mutant versions of UL42, in the activation of c-Jun/JNK. In particular, since the authors have previously generated and reported on a HCMV defective on UL42. Finally, the authors do not convincingly determine the region involved in c-Jun/JNK activation, as some conclusions are drawn from mutants with vast deletions, and in the case of UL42ΔCt, from a protein with stability problems. Therefore, the introduction of more subtle mutations in UL42 are recommended.

Other concerns:

- Figure 2B does not include clear pictures that support the conclusions drawn by the authors in the manuscript. It is intriguing why the UL42Ct has been generated as a GFP fusion protein and not as an HA-tagged-protein in the same way as WT UL42 and the rest of UL42 mutants. UL42Ct should be cloned in the HA-based vector and analyzed in this background so results are clearer and they could be directly compared with those obtained with the rest of constructs.
- The authors should have included the UL42Ct protein in the assays shown in Fig 1 and 3.
- Conclusions have been drawn based on the UL42ΔCt mutant. However, Fig 3 shows that the corresponding protein is not expressed in transfected cells, and the authors indicate that this might be due to the instability of the protein. It is hard to know what to make of it. The observation probably questions the results obtained with this mutant. In addition, if this is the case, why then the authors detect the expression of UL42ΔCt by immunofluorescence in Fig. 2A?
- In order to base conclusions from Fig 2A, images of some of the most relevant immunofluorescence pictures at a higher magnification should have been added.

Minor concerns:

- The Material and Methods miss information. For example, it does not contain immunoblot or fluorescence microscopy sections that could provide details on the conditions used in these procedures, such as lysis buffer, percentage of SDS-

PAGE, permeabilizing conditions, type of microscopy (is it confocal?), or magnification used in the study.

- The authors have previously reported that UL42 degrades Itch via the two PPXY motifs. However, and although they do not present a quantification of the normalized Itch band, this does not seem the be the case in the western blot shown in Figure 3. They should comment on this.
- Why in the western blot shown in Fig 3 UL42ΔN migrates as two bands?
- The information of the function recently attributed to UL42 (Fu et al. PLoS Pathogens 2019, 15(5): e1007691) should be mentioned in the Introduction too to contextualize the viral protein.
