# Supplementary material for: Activation of c-Jun by human cytomegalovirus UL42 through JNK activation
Source: PLoS One. 2020 May 5;15(5):e0232635. doi: 10.1371/journal.pone.0232635 (PMC7199950; doi:10.1371/journal.pone.0232635)
Supplement: Attachment — Submitted filename: PONE-D-20-02197 Response to Reviewers.docx [file pone.0232635.s007.docx]

**Responses to Reviewer’s comments**

**MS#:PONE-D-20-02197**

We thank you for your review on our manuscript. To accommodate your comments, we added a couple of experiments and modified the text, figures and supplementary materials as described below. Line numbers indicated by the reviewers are based on the original manuscript and those by us are based on the revised manuscript with tracked changes (file: PONE-D-20-02197 Revised Manuscript with Track Changes).

**Reviewer #1**

**General comment:** It is important to test whether UL42 is able to activate JNK during CMV infection.

**Response:**　To accommodate the comment, we conducted an additional experiment using recombinant HCMV strains with mutated versions of UL42 and found that HCMV infection induced c-Jun phosphorylation although the deletion of UL42 did not affect the c-Jun phosphorylation status in HCMV infected cells. These results could be explained by the presence of multiple mechanisms of the c-Jun phosphorylation in HCMV-infected cells. To describe our observation and discussion on this observation, we added immunoblot images as Figure S2 as well as sentences describing construction of recombinant HCMV strains (lines 143- 156), the results (lines 228-230) and discussion (lines 259- 261).

**Comment 1 (Figure 1)**: The reporter plasmid with pAP1 needs a detailed description. Also, the PPXY to PPXA alternation needs be clarified. Why UL42Ct start from 99aa rather than 87aa in the EGFP-UL42Ct mutant?
**Response:** We used a commercial reporter plasmid, pAP1(PMA)-TA-Luc, which contains multiple copies of the AP1 element and a minimal promoter, located upstream of the firefly luciferase gene. Descriptions of these details were added into the text (lines 98- 102).

Construction of the UL42PA mutant was described in our previous study (Koshizuka et al., 2016 J. Gen. Virol.).　 Briefly, tyrosine (Y) codons in the two PPXY (PY) motifs were replaced with alanine (A) codons by site-directed mutagenesis, resulting in the two PPXA (PA) motifs.　 We modified the text for clarification (lines 175-179).

Thanks for pointing out the error of the UL42Ct start site. EGPF-UL42Ct should contain amino acid residues from 87 to 124. Accordingly, Figure 1A was corrected.

**Comment 2 (Figure 2A)**: The way of tagging HA in mutants needs more detailed description. Why the signal intensity of c-Jun is different between vector, △Ct and WT? As is shown in Figure 3 its expression level is similar among the groups. A ratio between nuclear and cytoplasm c-Jun signal may be more convincing, and a statistic analysis is necessary.

**Response:** The details of construction of HA-tagged UL42 plasmids were added to the Materials and Methods section (lines 77-83).

As the reviewer noticed, although the amounts of c-Jun in the cells were at a similar level in immunoblotting, the signal intensities of c-Jun were different in spite of the use of the same anti-c-Jun antibody in both assays. We assume that the used antibody reacted better for nuclear c-Jun.

To accommodate the comment, the percentages of cells containing nuclear c-Jun among the cells expressing UL42WT or mutated versions were obtained for statistical analyses. Descriptions for the procedures and for the statistical results were added to the Materials and Methods section (lines 158-168) and to the Results section (lines 198-201 and 210-212), respectively. The statistically significances are also shown in the revised Figures 1B, 1C, 2B and 3B.

**Comment 3:** Figure 2B, it is necessary to add a positive group “EGFP-UL42wt” to rule out the possibility that EGFP has influence on intracellular localization of c-Jun.
**Response:** As suggested by the reviewer, we conducted an experiment that included EGFP-UL42WT and -UL42AY as controls (Figures 3 and 4 in the revised manuscript). Both EGFP-UL42WT and -UL42AY induced AP1 signaling and c-Jun nuclear accumulation and phosphorylation, which is consistent with the results using HA-tagged UL42 derivatives. To describe those results, the text was modified (lines 84-89, 181-185, 202-213, and 226-227).

**Comment 4**: Line 100-103. The conclusion ”These results indicate that the C-terminal region of UL42, but not the PY motif, is involved in the activation of AP-1-dependent transcription” may not be accurate, as shown in figure 3, C-terminal domain of UL42 is important for protein stability, so the low luciferase activity may be because of low protein expression level. Line113-114. The conclusion about C-terminal domain of UL42 may also not be accurate, because the protein expression level of UL42 is extremely low.

**Response:** In an additional experiment using EGFP-fusion proteins (revised Fig. 1C and Fig. 3), we found that the C-terminal transmembrane domain of UL42 alone did not accumulate c-Jun in the nuclei nor activated c-Jun. As criticized by the reviewer, the amounts of HA-tagged UL42ΔCt protein fluctuated from one experiment to another due to the stability issue. Taking account of the additional results and the stability issue, we removed the results obtained by expression of HA-tagged UL42ΔCt and modified our conclusion.

**Minor comment1:** line 93 “;” should be “:”
**Minor comment 2**: line105 “UL42wt, we” should be “UL42wt. We”
**Response:** As suggested, we corrected those errors (lines 114-118, and 181).

**Reviewer #2**

**General comment 1:**

However, a major problem of the study is the minimal amount of data presented, which in addition do not provide information on the mechanism by which UL42 activates c-Jun and JNK. Another major issue lies on the fact that all the observations described in the study are based on the UL42 protein ectopically expressed, and there are not indications that these UL42-mediated processes occur in the context of the HCMV infection. Although HCMV encodes additional proteins that activate the JNK pathway, it would seem important to determine the effect of a HCMV defective in UL42, or more specifically of HCMVs with mutant versions of UL42, in the activation of c-Jun/JNK. In particular, since the authors have previously generated and reported on a HCMV defective on UL42. Finally, the authors do not convincingly determine the region involved in c-Jun/JNK activation, as some conclusions are drawn from mutants with vast deletions, and in the case of UL42ΔCt, from a protein with stability problems. Therefore, the introduction of more subtle mutations in UL42 are recommended.

**Response:** As described in the response to Reviewer #1’s General comment, we conducted an additional experiment using recombinant HCMV strains and found that the lack of UL42 did not affect the c-Jun phosphorylation status in HCMV-infected cells. These results could be explained by the presence of multiple mechanisms of the c-Jun phosphorylation in HCMV-infected cells. We added the immunoblot images as Figure S2 and modified the text (lines 143-156, and 259-261).

In addition, we conducted addition experiments using plasmids expressing EGFP-tagged UL42 derivatives and found that both EGFP-UL42WT and -UL42AY activated AP-1 signaling. These observations were consistent with the results using the plasmids expressing HA-tagged proteins. At the same time, AP-1 activation, nuclear accumulation of c-Jun and c-Jun phosphorylation were significantly reduced in the cells expressing EGFP-UL42Ct. Therefore, it is likely that the C-terminal TMD was not involved in AP-1 signaling. We modified the text (lines 84-89, 181-185, 202-213, and 226-227) and revised Fig. 1C and Fig. 3. As criticized by the reviewer, the amounts of HA-tagged UL42ΔCt protein fluctuated from one experiment to another due to the stability issue. Taking account of the additional results and the stability issue, we removed the results obtained by expression of HA-tagged UL42ΔCt, and modified our conclusion.

As we agree with the need of experiments using more subtle mutations, we would like to address such a fine mapping in the next step. We added a description regarding this issue as one of limitations of this study (lines 257-258).

**Comment 1:** Figure 2B does not include clear pictures that support the conclusions drawn by the authors in the manuscript. It is intriguing why the UL42Ct has been generated as a GFP fusion protein and not as an HA-tagged-protein in the same way as WT UL42 and the rest of UL42 mutants. UL42Ct should be cloned in the HA-based vector and analyzed in this background so results are clearer and they could be directly compared with those obtained with the rest of constructs.
**Response:** To make UL42Ct data comparable with HA-tagged UL42 derivatives, we added a plasmid expressing EGFP-UL42WT as a positive control and a plasmid expressing UL42AY as a PPxY mutant in the revised Fig. 3. In addition, to show clear subcellular localization, the data of immunofluorescence assay at a higher magnification were added as Figure S1. Accordingly, the text was modified (lines 84-89, 181-185, 202-213, and 226-227)

**Comment 2:** The authors should have included the UL42Ct protein in the assays shown in Fig 1 and 3.
**Comment 3**: Conclusions have been drawn based on the UL42ΔCt mutant. However, Fig 3 shows that the corresponding protein is not expressed in transfected cells, and the authors indicate that this might be due to the instability of the protein. It is hard to know what to make of it. The observation probably questions the results obtained with this mutant. In addition, if this is the case, why then the authors detect the expression of UL42ΔCt by immunofluorescence in Fig. 2A?

**Response:** As described for Comment 1, we added EGFP-UL42WT and UL42AY to compare with EGFP-UL42Ct and the C-terminal TMD of UL42 was not involved in AP-1 activation. Taking account of the new results, we modified our conclusion.

**Comment 4:** In order to base conclusions from Fig 2A, images of some of the most relevant immunofluorescence pictures at a higher magnification should have been added.
**Response:** As suggested, we added the immunofluorescence assay images at a higher magnification as Figure S1.

**Minor concerns:**

**Comment 5:** The Material and Methods miss information. For example, it does not contain immunoblot or fluorescence microscopy sections that could provide details on the conditions used in these procedures, such as lysis buffer, percentage of SDS-PAGE, permeabilizing conditions, type of microscopy (is it confocal?), or magnification used in the study.

**Response:** As suggested by the reviewer, we intensively modified the Materials and Methods section (lines 67-168).

**Comment 6:** The authors have previously reported that UL42 degrades Itch via the two PPXY motifs. However, and although they do not present a quantification of the normalized Itch band, this does not seem to be the case in the western blot shown in Figure 3. They should comment on this.

**Response:** In order to show more clear results, the immunoblot image of Itch in Figure 4A was revised. As shown in Figure 4A, Itch was decreased in UL42WT and ΔI expressing cells but not in UL42PA or ΔN expressing cells.

**Comment 6:** Why in the western blot shown in Fig 3 UL42ΔN migrates as two bands?

**Response:** Based on the molecular weights of the two bands, we assume that they are products of proteolytic cleavage (lines 218-219).

**Comment 7:** The information of the function recently attributed to UL42 (Fu et al. PLoS Pathogens 2019, 15(5): e1007691) should be mentioned in the Introduction too to contextualize the viral protein.

**Response:** As suggested by the reviewer, we described the study in the Introduction section (lines 56 – 59).

**Reviewer #3**

**General comment:** Although the work is technically sound in most places, revisions must be made before the conclusions drawn by the authors are fully supported by the data. One area of concern is Figure 1B, where it is stated that “ratios… obtained in triplicated wells are shown as the mean ±SEM” suggesting that data is from a single experimental repeat. Can this result be reproduced and could the authors show these results as the mean ±SEM from across multiple experimental replicates?

**Response:** Our luciferase assay results are based on three independent experiments and each set of experimental results was analyzed with the one-way ANOVA test followed by the Tukey’s comparison test using GraphPad PRISM software. The data obtained in triplicated wells of one of three independent experiments are presented as Figure 1B and C. The text were revised for clarification (lines 159-168 and 372-380).

**Comment 1:** In Figure 2, the authors show that their EGFP-UL42Ct construct does not induce nuclear localization of c-Jun. This is a very interesting and important result. Could the authors therefore also show that this construct does not activate Jun/JNK/AP-1 either by luciferase assay as in figure 1, and/or by immunoblot as in figure 3? This is not vital for the integrity of the manuscript but would greatly enhance the claims being made.

**Response:** To accommodate this comment, EGFP-UL42WT and UL42AY were added in this manuscript as controls for EGFP-UL42Ct. AP-1 signal was activated and c-Jun was accumulated to nucleus by expression of EGFP-UL42WT and -UL42AY but not by EGFP-UL42Ct. To describe these results, the text was modified (lines 84-89, 181-185, 202-213, and 226-227) and revised Fig. 1C and Fig. 3.

**Comment 2:** In Figure 3, the authors were unable to detect UL42∆Ct by immunoblot and conclude that UL42∆Ct is too unstable to be detected. This raises problems with their results in Figure 1, as they do not demonstrate that UL42∆Ct was expressed by the cells in this assay. If UL42∆Ct was not being expressed, then the authors cannot conclude that the C terminal TMD is required for UL42-mediated activation of AP-1.
**Comment 3:** However, in Figure 2A the authors can detect UL42∆Ct by immunofluorescence assay. Figure 2A therefore suggests that UL42∆Ct is expressed just as well as other UL42 mutants. This indicates that there is potentially a problem with the immunoblotting protocol that is used to solubilize and detect UL42 in Figure 3. Since UL42∆Ct is no longer a membrane protein, it could be that a different immunoblotting approach is required to detect it. It is difficult to suggest what other problems may be causing this as the authors have not included a detailed explanation of their immunoblotting protocol in the methods section.

**Response:** As described for the comments of Reviewers #1 and #2, we conducted addition experiments using plasmids expressing EGFP-fusion proteins and found that both EGFP-UL42WT and -UL42AY activated AP-1 signaling. These observations were consistent with the results using the plasmids expressing HA-tagged proteins. As in the same experiments, AP-1 activation and c-Jun phosphorylation were significantly reduced in the cells expressing EGFP-UL42Ct, it is likely that the C-terminal TMD was not involved in AP-1 signaling. We modified the text (lines 84-89, 181-185, 202-213, and 226-227) and revised Fig. 1C and Fig. 3. As criticized by the reviewer, the amounts of HA-tagged UL42ΔCt protein fluctuated from one experiment to another due to the stability issue. Taking account of the additional results and the stability issue, we removed the results obtained by expression of HA-tagged UL42ΔCt and modified our conclusion.

**Comment 4:** Figure 2 provides strong evidence to support the conclusions drawn by the authors. I believe that the improvements to the data in Figures 1 and 3 that I have stated so far would strengthen the rigor of this manuscript sufficiently to justify the author’s conclusions.
**Response:** To accommodate the comment, we added the statistical analysis in Figures 1 and 2. In addition, we added EGFP-UL42WT and -UL42AY to compare EGFP-UL42Ct (revised Fig. 3).

**Comment 5:** Has the statistical analysis been performed appropriately and rigorously?
**Comment 6:** Statistical analysis, ideally using one-way analysis of variance (ANOVA), needs to be performed for luciferase data in Figure 1B in order to calculate the statistical significance of the results presented. This analysis should be performed on data from three experimental repeats.

**Response:** As suggested, we conducted statistical analyses using GlaphPad PRISM software. All statistical analysis was performed on data from three independent experiments and we confirmed that these data were significant statistically. The text was revised (lines 158-168, 374-378, 388-392, and 398-401).

**Comment 7**: Is the manuscript presented in an intelligible fashion and written in standard English?

**Response**: Our manuscript was edited commercially by an English-native scientific editor.

**Comment 8-1:** The abbreviations (PA) and (∆N) are not defined in the text.

**Response:** Sentences describing the definitions of PA and ∆N were added to the text (lines 79-83, and 179-181).

**Comment 8-2:** A control vector is used, but it is labeled as “V” in Figure 1, “Vector” in Figure 2 and “Vec” in Figure 3. The authors should state if these are the same vector, and what this vector is (I assumed that it is the empty UL42 expression vector, pCAGGS).

**Response:** We modified the legends for these figures.

**Comment 8-3**: Is anything already known about the internal region or C terminal TMD? If so, this should be detailed in the introduction (or stated that nothing is known).

**Response:** As there are no additional information about the domain mapping of UL42, the text was revised for clarification (lines 49-52).

**Comment 8-4:** When the authors discuss the proximal regions of the C-terminal TMD, is this the same region as the internal region, or are they different? The authors could label Figure 1A to help clarify which regions of UL42 they are discussing.

**Response:** For clarification, the sentence was revised (line 212-213, 246-249, and 275-276).

**Comment 8-5:** The figure legends for figures 1 and 2 need to include definitions for the UL42 mutants, as in figure 3.

**Response:** The legends were revised (lines 378-380, 383-387, and 398-400).

**Comment 9:** There is no mention in the methods section of how immunoblots were performed, a detail which is important for understanding the lack of expression of the ∆Ct mutant in Figure 3. Other details that should be included in the methods section:

- Antibody concentrations for immunofluorescence and immunoblotting
- Antibody incubation conditions
- Enzymes and ligases used for cloning UL42 into the pCAGGS vector would be useful
- The amount of each plasmid that was transfected for each assay

**Response:** The Materials and Methods section were intensively revised to include these information. (lines 76-95, 97-104, 106-118, and 120-141)

**Others**

1. To increase readability, the text was edited without losing the original contents.

2. Primers names were simplified.
