# Supplementary material for: Activation of c-Jun by human cytomegalovirus UL42 through JNK activation
Source: PLoS One. 2020 May 5;15(5):e0232635. doi: 10.1371/journal.pone.0232635 (PMC7199950; doi:10.1371/journal.pone.0232635)
Supplement: Attachment — Submitted filename: 1 PONE-D-20-0219R1 Response to Reviewers.docx [file pone.0232635.s008.docx]

**Responses to Reviewer’s comments**

**MS#:PONE-D-20-02197R1**

We thank you for your review on the revised version of our manuscript. To accommodate your comments, we modified the text and supplementary materials as described below. Line numbers indicated by the reviewers are based on the revised manuscript and those by us are based on the re-revised manuscript with tracked changes (file: PONE-D-20-02197R1 Revised Manuscript with Track Changes).

**Reviewer #1**

**Comment 1 (Figure 1)**: It is good to use HA and GFP to rule out the effects of tag protein on results. But the experiments control from this two different tag groups should keep consistent. Tagged-UL42WT, -UL42N, -UL42I, -UL42PY, -UL42AY, and -CT should be included in both two groups.

**Response:** We used EGFP-UL42Ct to confirm the requirement of the UL42 C-terminus region for activation of c-Jun/JNK signaling. On the other hand, the EGFP-UL42AY mutant was used for a separate issue, that is, confirmation of no involvement of the Nedd4 family E3 ligases in the c-Jun/JNK signaling. The text was revised to clarify the respective purposes of these constructs (lines 181-185).

**Comment 2:** Line226-227. ”c-Jun and JNK were phosphorylated by the expression of EGFP-UL42WT and -UL42AY but not by EGFP-UL42Ct.” But as Figure 4B shows, the phosphorylation level of c-Jun by EGFP-UL42Ct is almost the same as EGFP-UL42WT and UL42AY and the ph9osphorylation level of JNK by EGFP-UL42Ct is nearly to EGFP-UL42AY. More experiment controls need to be set to explain this problem or the conclusion need to be amended.

**Response:** Although the phosphorylation level of c-Jun seems weak in EGFP-UL42WT and -AY expressing cells, the phosphorylation of JNK was significantly increased in EGFP-UP42WT or -AY expressing cells but not in EGFP-UL42Ct expressing cells (Fig.4B). In addition, the results of luciferase assay (Fig.1C) and nuclear translocation of c-Jun (Fig.3) supported the notion that the phosphorylation of c-Jun and activation of AP-1 signaling occurred by expression of EGFP-UL42WT or -AY but not by expression of EGFP-UL42Ct. To accommodate the reviewer’s comment, we added some sentences discussing the issue (lines 246-253)

**Comment 3** : Line 275-276. As comment 2 mentioned, taken all results into consideration, aa52-86 region of UL42 is just responsible for Jun translocation to nucleus.

**Response:** We agree. To accommodate the comment, we modified the text (lines 298-299)

**Minor comments:**

1. line247-248 “c-Jun” should be “C-Jun”
2. Line246-247 needs rephrasing.
3. Line184-185, this sentence is confusing, according to the results, “except for” should be “rather than”

**Response:** As suggested, these sentences were corrected or rephrased (lines 196, 202, and 230 for #1, lines 256-259 for #2, and lines 185-189 for #3).

**Reviewer #2**

**Minor comment 1:**
The legend to Fig S2 needs to be revised. It is not correct to state "Fibroblasts infected with mock (m) ..."
**Response:** As suggested, the sentence was corrected (lines 465-471).

**Reviewer #3**

**Comment 1:** Figure 1B shows the results from one of three experiments, including statistical analysis. Could the authors include the data for the other two experiments, either as mean averages in Figure 1B, or at least in the supplemental section as two separate figures?
**Response:** As suggested, we revised the sentences describing that the results of Figure 1B and 1C indicated the means of three independent experiments. In addition, we added a supplementary figure (S1 Figure) showing the three independent sets of results (lines 103-105 and 401-404, and 445-453).

**Comment 2:** In Figure 2B, could the authors include the empty vector control in their quantification of Jun positive nuclei? Could the authors also label the y axis to describe what this graph shows? Similarly for Figure 3B, the y axis needs a label.

**Comment 3:** Figure S1A requires an empty vector control.

**Response:** Figure 2B presented the percentage of nuclear c-Jun positive cells in cells expressing HA-UL42 or its variant. It is technically hard to use the empty vector (pCAGGS) for the quantification, as it does not express detectable HA-tagged protein, which is a situation different from that of the empty vector for EGFP fusion. In the same context, it is impossible to add an image with an empty vector control in Figure S1A, as we have no way to distinguish cells transfected with the empty vector from those not transfected. As suggested, the labels of y axis were added to Figures 2B and 3B.

**Minor comment 1:** The sentence in results section lines 181-185 does not make sense. I think that removing the word "and" so that this reads: "...the PY motifs is essential for the activation of AP-1 dependent transcription" is what the authors want to say.

**Response:** As suggested, we corrected the sentence (lines 185-189).

**Minor comment 2:** In figures 3A and 4B, EGFP-Ct is detectable by immunofluorescence but not by western blot. The authors suggest that the difference in their observation is due to variable protein stability. Having examined figures 3A and S1B, it does appear as if the EGFP-UL42Ct construct has weaker fluorescence than the other constructs. Would the authors agree with this, as it would then make their results consistent? If so, could the authors mention this weaker fluorescence in the discussion.

**Response:** We agreed the reviewer’s comment. We pointed out the weaker signals in the Result section and discussed the issue (lines 260-268).

**Minor comment 3:** The final paragraph of the results section describes the infection assay performed by the authors. Could the authors include details such as cell type infected, multiplicity of infection and the duration of infection? This information is vital to analyzing this data and should be mentioned in the results as well as the figure legend. Perhaps, for example, if the authors have infected these cells at a higher MOI (higher than 1) this could lead to significant activation of Jun simply due to the large amount of virus present. Using a lower MOI may therefore reveal differences between WT and UL42 deletion viruses for Jun activation. Without this information, it is hard to determine the validity of this result.

**Response:** As requested, we added the details of infection conditions in the legend for Figure S3 of this revised manuscript (lines 465-471).

**Minor comment 4:** It is not surprising that there was no difference between WT and the UL42 deletion mutant for Jun activation, given that other HCMV genes also activate Jun, as stated by the authors. As I understand it, Dunn et al showed in 2003 that UL42 is dispensable for infection in fibroblasts. This should be mentioned by the authors as it supports the notion that UL42's function is redundant during infection.

**Response:** As suggested, a sentence describing the previous studies with citations, including Dunn et al, was added (lines 290-292).

**Minor comment 5:** As the transfection assays were performed in HEK293T cells, and the infections performed in fibroblasts, could activation of Jun by UL42 be cell-type specific? The authors could either perform transient transfection assays in fibroblasts to resolve this difference, or simply moderate their discussion section to specify that this observation is in transfection assays of HEK293T cells and not fibroblasts.

**Response:** It is very important point, but we did not determine the cell type specificity of the activation of AP-1 signaling by UL42. As the fibroblast cells which we used have very low transfection efficiency, it is difficult to analyze the effects quantitatively. As suggested, we clarified that the observation was in HEK293T cells and addressed the issue in Discussion (lines 276-282).

**Others:**

We rephrased “293T cells” as “HEK293T cells”.
